# Evidence for Treatment-by-Biomarker interaction for FDA-approved Oncology Drugs with Required Pharmacogenomic Biomarker Testing

**DOI:** 10.1038/s41598-017-07358-7

**Published:** 2017-07-31

**Authors:** Alexandre Vivot, Isabelle Boutron, Geoffroy Béraud-Chaulet, Jean-David Zeitoun, Philippe Ravaud, Raphaël Porcher

**Affiliations:** 1 Clinical Epidemiology Unit, Hôtel-Dieu Hospital, Greater Paris University Hospital (AP-HP), Paris, France; 2 Epidemiology and Statistics Sorbonne Paris Cité Research Center (CRESS), INSERM, Paris Descartes University, Paris, UMR1153 France; 3 School of Medicine, Paris Descartes University, Sorbonne Paris Cité Paris, France; 4 Gastroenterology and Nutrition Department, Saint-Antoine Hospital, Greater Paris University Hospital (AP-HP), Paris, France; 5 Proctology Department, Croix Saint-Simon Hospital, Paris, France; 6 Department of Epidemiology, Mailman School of Public Health, Columbia University, New York, NY USA

## Abstract

For oncology drugs that were approved by the US Food and Drug Administration (FDA) and required pharmacogenomic biomarker testing, we describe 1) the use of enrichment (biomarker-positive patients) and a randomized controlled design by pre-approval trials and 2) the treatment-by-biomarker interaction. From the 137 drugs included in the FDA table, we selected the 22 oncology drugs with required genetic testing in their labels. These drugs corresponded to 35 approvals supported by 80 clinical studies included in the FDA medical officer reviews of efficacy. For two thirds of approvals (24/35, 69%), all clinical studies were restricted to biomarker-positive patients (enriched). Among the 11 remaining approvals with at least one non-enriched trial, for five approvals, the non-enriched studies were non-randomized. The treatment-by-biomarker interaction was statistically significant for three approvals and missing for two. Among the six approvals with a non-enriched randomized controlled trial, three featured a statistically significant treatment-by-biomarker interaction (p < 0.10), for an enhanced treatment effect in the biomarker-positive subgroup. For two thirds of FDA approvals of anticancer agents, the requirement for predictive biomarker testing was based on clinical development restricted to biomarker-positive patients. We found only few cases with clinical evidence that biomarker-negative patients would not benefit from treatment.

## Introduction

Anticancer agents are increasingly being combined with a biomarker to determine which patients are the most likely to benefit from the drug. Examples are vemurafenib combined with BRAF V600E mutation or cetuximab with RAS mutation^1–3^. As acknowledged by drug regulators in the European Union and the United States, a drug approval supported only by trials restricted to biomarker-positive patients (ie, enriched trials) would be logically indicated for the biomarker-positive subpopulation only, but this restriction does not indicate that the biomarker is a good one.

According to the CDC-sponsored Evaluation of Genomic Applications in Practice and Prevention (EGAPP) working group, validation of a biomarker requires four dimensions of evidence: 1) the analytic validity (the technical performance of the test); 2) the clinical validity (the test’s ability to diagnose a disorder, assess susceptibility or risk, or provide information on prognosis or variation in drug response); 3) the clinical utility (evidence that test results can change patient management decisions and improve net health outcomes); and 4) the ethical, legal, and social implications of the use of the biomarker^4^. There are diverging opinions among different stakeholders and scientific societies on what constitutes clinical validity and utility for a predictive biomarker and how to assess the supporting evidence^4–7^. Overall, the clinical validity relates to the ability of the biomarker to predict response to the treatment, whereas its clinical utility would ideally be a direct consequence of the clinical validity^6, 8, 9^. A lack of common evidentiary standards of clinical utility of biomarker testing for predictive biomarkers has been identified as a barrier to precision medicine^10–12^.

However, a central idea widely shared among stakeholders is the reliance on randomized controlled trial (RCT) data in assessing clinical validity and utility because with single-arm studies, the prognostic effect of the biomarker (biomarker-positive patients may have better outcome regardless of the treatment received) cannot be distinguished from the predictive effect (the treatment effect is higher in biomarker-positive than -negative patients)^6–9, 13–15^. Another point—maybe even more central—is that a qualitative treatment-by-biomarker interaction is at the core of precision medicine, as underlined in the prognosis research strategy (PROGRESS) paper on stratified medicine^6–9, 13–15^. Indeed, a predictive biomarker should define one subgroup with a positive (beneficial) treatment effect (who should receive treatment) and another with a non-positive treatment effect (who should not receive treatment)^15^. This has been recognized by many authors including in some publications by FDA officials that “*The most straightforward way to establish that a biomarker is predictive is to demonstrate a statistically significant interaction between treatment and biomarker status in the context of the analysis used to demonstrate an overall treatment effect”* ^16^.

According to a group of diverse stakeholders convened by the Center for Medical Technology Policy, the preferred design to evaluate the clinical utility of a predictive biomarker is the all-comer prospective marker-stratified design, whereby all patients (biomarker-positive and -negative) are randomized between the experimental and control arms and the randomization is stratified on the biomarker status^9^. This design indeed allows for a proper evaluation of the treatment-by-biomarker interaction. However, because stratifying the randomization on the marker is sometimes difficult (for logistic reasons or obviously if the biomarker is discovered after the launch of the trial), a prospective-retrospective design can be used^12, 16, 17^. A prospective-retrospective RCT includes a biobank for performing biomarker-defined subgroup analyses after the completion of the trial. Under some conditions (e.g., pre-specification of the analysis plan, low proportion of tumors with failed marker testing), this design can lead to the same high level of evidence for clinical utility as the prospective marker–stratified design.

Besides differences between fully prospective and prospective-retrospective trials, personalized medicine needs randomized controlled trials^18^. Indeed, to have a complete picture of the efficacy of a drug with a predictive biomarker, ideally, we need data on the treatment effect in both biomarker-positive and -negative patients. However, in some cases, preclinical data or an understanding of the mechanism of action of the drug is sufficient and clinical data for biomarker-negative patients are not needed. Hence, exposure of biomarker-negative patients to the experimental drug would be unethical. In this case, the preferred design is the enriched design, enrolling only biomarker-positive patients^12^. Even in this situation, we still need RCTs to be able to estimate the treatment effect. A biomarker could be naturally binary, such as the presence of a gene mutation (wild-type vs mutated), or a continuous marker that has been dichotomized, such as the expression of a protein. By biomarker-positive, we mean the biomarker-defined subgroup of patients thought to have a greater magnitude of treatment effect.

In this study, for oncology drugs that were approved by the US Food and Drug Administration (FDA) and required pharmacogenomic biomarker testing, we aimed to describe 1) the use of enrichment and a randomized controlled design by pre-approval trials and 2) the treatment-by-biomarker interaction.

## Methods

### Source of data

From the FDA Table of Pharmacogenomic Biomarkers in Drug Labeling^19^, we identified the drug–biomarker pairs with required genetic testing in the drug label that were indicated for oncology as previously described^20^. We used the most recent label (as of June 10, 2015) to search for all indications of a drug. We did not include indications for which the biomarker was not relevant and non-oncology indications. We also excluded indications for which the proportion of biomarker-positive patients was very high (Supplementary Table S1) acknowledging that, in these cases, conducting studies with a sufficient number of biomarker-negative patients to demonstrate a treatment-by-biomarker interaction would be unfeasible. Example diseases included chronic myeloid leukemia (CML), which is almost always the result of a genetic translocation (Philadelphia chromosome)^21, 22^.

For each drug indication, we extracted the list of clinical studies on which the FDA bases its approval decision from the FDA medical review. When this review was not available, we used the content of the approval letter and the drug label. We included studies relevant to the indication only and excluded studies reviewed for only safety data as well as pharmacology and ongoing studies.

### Extraction of clinical trial characteristics

We extracted the name of the biomarker, if any, used for restricting trial entry based on biomarker status. We also extracted whether the trial was randomized; whether the FDA considered the trial as a pivotal trial (trials that the FDA relies on for its approval decision, usually a phase III controlled trial with a large sample size) and trial general characteristics. For each included trial, clinical trial characteristics were manually extracted by two investigators (AV and GBC) who used a standardized extraction form, and any discrepancies were resolved by discussion.

### Availability of treatment-by-biomarker interaction

We first examined for each indication if there was at least one non-enriched trial (ie, enrolled both biomarker-positive and -negative patients). For indications for which all trials were conducted with biomarker-positive patients, no treatment-by-biomarker interaction was available. In such cases, we considered that the clinical validity and utility was exclusively based on mechanistic hypotheses and preclinical data. Then, for indications with at least one non-enriched trial, we separated single-arm and randomized trials. Finally, for indications with at least one non-enriched randomized clinical trial, we assessed the statistical significance of the treatment-by-biomarker interaction. Two reviewers (AV and GBC) independently assessed the characteristics of evidence, with consensus with a third investigator (RP).

### Post-marketing studies

Because evidence for the treatment-by-biomarker interaction evolves after the drug approval, especially for indications granted within the accelerated approval pathway, we searched for post-marketing studies related to the biomarker by searching letters issued by the FDA to the sponsor and the online FDA database for post-marketing commitments^23^. We also examined the CLINICAL STUDIES section of all versions of the drug label to identify potential studies added after the approval.

### Statistical analysis

When not reported in the original paper, we extracted data needed to compute the treatment-by-biomarker interaction test and we computed the p-value for the interaction by a Z-test to compare two log hazard ratios (HRs) for survival endpoints or two odds ratios for binary endpoints^24^; 95% confidence intervals were estimated. For single-arm trials, we computed a p-value for differences in outcome for biomarker-negative and -positive patients by using Fisher’s exact test for binary endpoints. Because interaction tests are generally underpowered^25^, we used a significance level of p < 0.10. All analyses involved use of R v3.1 (R Core Team, Vienna, Austria)^26^.

### Ethical Statement

All methods were carried out in accordance with relevant guidelines and regulations. No informed consent or ethics approval was necessary because this study is based on publicly available data and involved no individual patient data collection or analysis.

### Availability of materials and data

See Tables, Figures, and supplemental files for the data supporting the statement in this article. All these data were extracted from publicly available US FDA’s website and documents.

## Results

### Drugs approved

**Figure 1:**
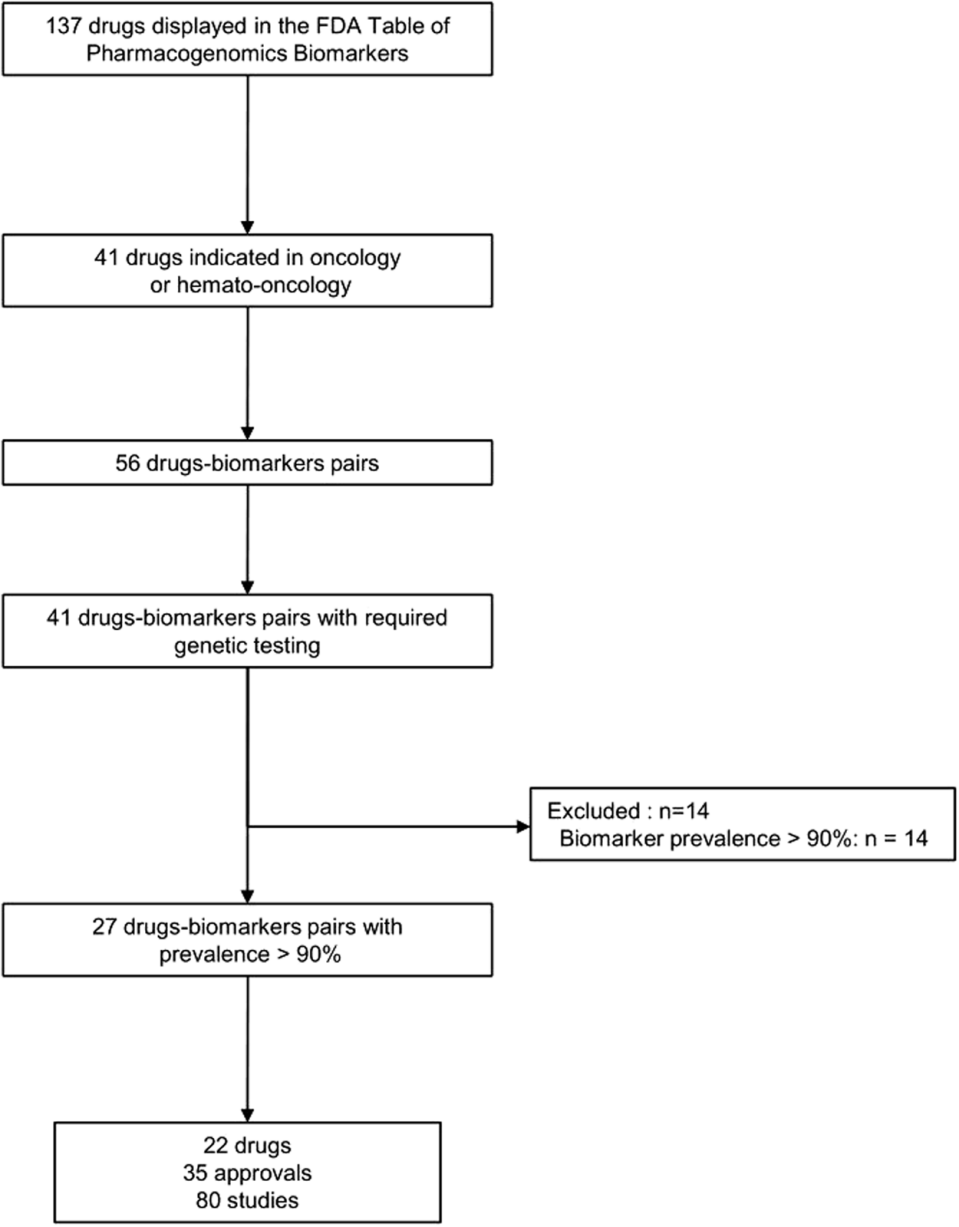
Flow of drugs, approvals and clinical trials included in the current study.

From the 137 drugs included in the FDA table, we selected the 22 oncology drugs with required genetic testing in their labels (Fig. 1). All were targeted drugs according to the US National Cancer Institute list^27^ and represented 27 drug–biomarker pairs and 35 indications (or approvals), Table 1. Nine approvals (26%) had been granted with orphan drug status, and 12 (34%) via the accelerated approval pathway. Seventeen approvals (49%) were original approvals and the remaining were supplemental approvals (new or modified indication).

**Table 1:** List and Main Characteristics of Included FDA Drug Approvals (n = 35).

Drug	Biomarker gene	Indication	Original (O) or supplemental (S) approval	Accelerated Approval or regular	No. of trials	No. of non-enriched trials*	No. of RCTs	No. of Trials with OS/PFS	No. of patients enrolled in trials
Ado-Trastuzumab Emtansine	ERBB2	Breast cancer	O	regular	4	0	1	2	1237
Afatinib	EGFR	Lung cancer	O	regular	6	0	2	6	2329
Anastrozole	ESR1, PGR	Breast cancer (early stage)	S	accelerated	1	1	1	1	9366
Anastrozole	ESR1, PGR	Breast cancer (advanced stage)	S	regular	1	0	1	0	668
Cetuximab	EGFR	Colorectal cancer	O	accelerated	3	0	1	3	525
Cetuximab	KRAS	Colorectal cancer	S	NA	5	5	5	5	4141
Crizotinib	ALK	Lung cancer	O	accelerated	2	1	0	0	302
Dabrafenib	BRAF	Melanoma (single agent)	O	regular	3	0	1	3	514
Dabrafenib	BRAF	Melanoma (with trametinib)	S	accelerated	1	0	1	0	162
Dasatinib	BCR/ABL1	Blood cancer	O	accelerated	2	0	0	0	46
Denileukin Diftitox	IL2RA	Other	O	accelerated	2	0	1	0	106
Erlotinib	EGFR	Lung cancer	S	regular	1	0	1	1	173
Everolimus	ERBB2	Breast cancer	S	regular	1	0	1	1	724
Everolimus	ESR1	Breast cancer	S	regular	1	0	1	1	724
Exemestane	ESR1	Breast cancer	S	regular	1	1	1	1	4724
Fulvestrant	ESR1	Breast cancer	O	regular	2	2	2	2	924
Imatinib	KIT	Blood cancer	S	regular	2	2	0	0	30
Imatinib	BCR/ABL1	Blood cancer (Ph + ALL, adult patients)	S	regular	2	0	0	0	55
Imatinib	BCR/ABL1	Blood cancer (Ph + ALL, Pediatric patients in combination with chemotherapy)	S	accelerated	3	0	0	3	64
Imatinib	PDGFRB	Blood cancer	S	regular	2	2	0	0	31
Lapatinib (with capecitabine)	ERBB2	Breast cancer	O	regular	3	0	1	1	631
Lapatinib (with letrozole)	ERBB2	Breast cancer	S	accelerated	1	1	1	1	1286
Letrozole	ESR1, PGR	Breast Cancer (early stage)	S	accelerated	1	0	1	1	8010
Letrozole	ESR1, PGR	Breast cancer (advanced stage)	O	regular	3	0	3	1	2013
Panitumumab	EGFR	Colorectal cancer	O	accelerated	1	0	1	1	463
Panitumumab	KRAS	Colorectal cancer	S	regular	2	2	2	2	1292
Pertuzumab	ERBB2	Breast cancer (Metastatic)	O	regular	2	0	1	2	903
Pertuzumab	ERBB2	Breast cancer (Neo-adjuvant)	S	accelerated	2	0	2	0	642
Trametinib	BRAF	Melanoma	O	regular	3	1	1	3	516
Trastuzumab	ERBB2	Metastatic Breast cancer	O	regular	7	0	1	0	1163
Trastuzumab	ERBB2	Breast cancer, adjuvant	S	regular	2	0	2	2	3987
Trastuzumab	ERBB2	metastatic gastric adenocarcinoma	S	regular	1	0	1	1	594
Vemurafenib	BRAF	Melanoma	O	regular	3	0	1	1	856
Ceritinib	ALK	Lung cancer	O	accelerated	2	0	0	0	266
Lenalidomide	del (5q)	Blood cancer	O	regular	2	1	0	0	191

### Characteristics of clinical trials

The characteristics of the 80 clinical studies supporting the 35 approvals are in Supplementary Table S2 and the characteristics of the 40 pivotal studies are in Supplementary Table S3. The median (interquartile range [IQR]) number of studies per approval was 2 (1–3). Many approvals (27/35, 77%) were supported by at least one randomized clinical trial. The median (IQR) number of total patients enrolled in trials per approval was 642 (229–1262). For most of indication with regular approvals (16/22, 73%) there was at least one clinical trial with OS or PFS data.

### Evidence for treatment-by-biomarker interaction

**Figure 2:**
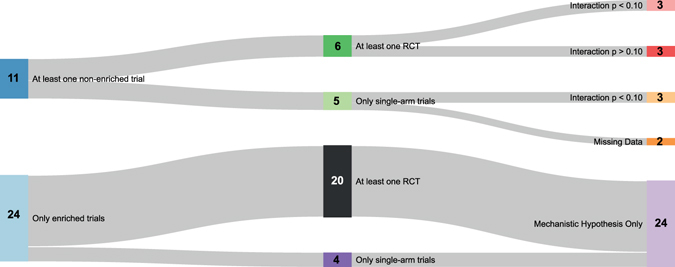
Sankey diagram representing the distribution of FDA drug approvals for which all trials included biomarker-positive patients (ie, enriched), the number of approvals with at least one non-enriched randomized controlled trial and the statistical significance of the treatment-by-biomarker interaction. Width of links is proportional to the number of approvals.

The Sankey diagram in Fig. 2 shows the distribution of indications for which all trials were enriched and for those with at least one non-enriched RCT, the statistical significance of the treatment-by-biomarker interaction. For two thirds of approvals (24/35, 69%), all clinical studies were restricted to biomarker-positive patients.

**Figure 3:**
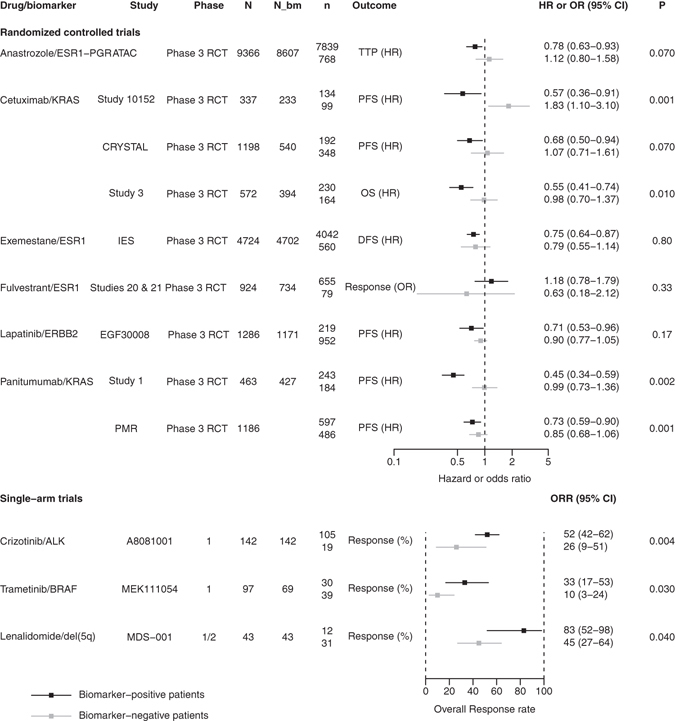
Characteristics of evidence supporting the clinical utility of pharmacogenomic biomarkers and treatment effect for 9 approvals of oncology drugs with at least one non-enriched clinical trial. Treatment effects are represented for biomarker-positive patients and biomarker-negative patients. *N* represents the total number of patients enrolled in the trial, *N_bm* the number of patients with a known biomarker status and *n* the number of patients in each biomarker-based subgroup. For randomized controlled trials, P is the p-value of the treatment-by-biomarker interaction. For single-arm trial, P is the p-value of the test comparing outcomes in biomarker-positive and biomarker-negative patients. Regarding fulvestrant/ESR1,when the superiority objectives were not met, the two pivotal trials were retrospectively assessed for non-inferiority with a 10% margin for overall response rate. Regarding Lapatinib/ERBB2, this approval is for the following indication: *“in combination with letrozole for the treatment of postmenopausal women with HR* + *Metastatic breast cancer expressing HER2 receptor for whom hormonal therapy is indicated*”. Regarding Crizotinib/ALK, a cohort of ALK-negative patients was added to study B (A8081001), which had enrolled 119 ALK-positive patients (105 evaluable by an independent committee review).

Among the 11 remaining approvals with at least one non-enriched trial, for five, the non-enriched studies were non-randomized and the treatment-by-biomarker interaction is shown in Fig. 3. However, because of the non-randomized design, this interaction only shows a differential response that mixes the prognostic and predictive effects of the biomarker. In two cases, (imatinib/KIT for aggressive systemic mastocytosis and imatinib/platelet-derived growth factor receptor B for myelodysplastic syndrome–myeloproliferative disease), the drug approval was supported by only case reports, so data on the interaction were not available. For six approvals, non-enriched studies were randomized and the treatment-by-biomarker interaction is shown in Fig. 3. With the randomized design, these interactions represent the predictive effect of the biomarker that is the differential treatment effect according to the biomarker status. For three approvals, the treatment-by-biomarker interaction was statistically significant (p < 0.10).

The three cases with significant treatment-by-biomarker interaction in an RCT were cetuximab/KRAS and panitumumab/KRAS, both for metastatic colorectal cancer; and anastrozole/hormonal receptor for adjuvant treatment of early breast cancer. For example, cetuximab was approved for metastatic colorectal cancer based on a phase III RCT that enrolled all patients and retrospectively assessed tumor biopsies for KRAS mutations^28, 29^. Treatment was effective for patients with KRAS wild-type tumors (progression-free survival HR = 0.57; 95% CI, 0.36–0.91) but not ﻿for those with KRAS-mutated tumors (HR = 1.83; 95% CI, 1.10–3.06, p(interaction) = 0.001).

An example of a non-significant treatment-by-biomarker interaction in the pivotal RCT is lapatinib, for treating metastatic breast cancer that overexpresses the human epidermal growth factor receptor 2 (HER2), combined with letrozole. Data supporting this indication were derived from a double-blind RCT. Among 1286 randomized patients, 219 (17%) were HER2-positive and benefited from lapatinib (disease progression HR = 0.71; 95% CI, 0.53–0.96), with no benefit demonstrated for HER2-negative tumors (HR = 0.90; 95% CI, 0.77–1.05)^30^. However, we found no evidence of a treatment-by-biomarker interaction (p = 0.17).

Examples of approvals with only single-arm non-enriched studies are crizotinib and trametinib. Crizotinib obtained accelerated approval in 2011 for treating metastatic non–small-cell lung cancer (NSCLC) based on two single-arm studies restricted to patients positive for anaplastic lymphoma kinase (ALK). Study A (NCT00932451) was an open-label, single-arm, multinational, phase II trial of 148 patients with ALK-positive NSLCC. Study B (NCT00585195) was an open-label, multicenter, multinational, phase I trial that included a dose-escalation cohort and a recommended phase II dose-expansion cohort. Following the FDA’s recommendation, a cohort of 23 ALK-negative patients was added to study B^31^. The response rate in these patients was surprisingly high considering that crizotinib was thought not effective in ALK-negative patients. Indeed, 5 of 19 evaluable patients (26%, 95% CI, 9–51%) achieved an investigator-assessed response rate and 2 (11%) had stable disease^31^. The response rate for ALK-positive patients was 52% (95% CI, 42–62%). We present data from this trial (A8081001) in Fig. 3. These two response rates were statistically different (p = 0.004). The indication was restricted to patients with ALK-positive NSCLC, but the FDA asked the sponsor to study crizotinib in ALK-negative patients in a post-marketing trial.

Trametinib was approved in 2013 for treating unresectable or metastatic melanoma. One phase 1 trial was not enriched, and data from this trial are in Fig. 3. Here again, based on the response rate in biomarker-negative patients, the FDA asked the applicant to enroll BRAF wild-type patients in the pivotal phase III RCT (MEK114267) but left the decision to the applicant, who decided not to follow this recommendation, and the pivotal phase III RCT was restricted to BRAF-mutated patients.

### Post-marketing studies

The FDA requested a post-marketing study (PMS) to investigate the biomarker validity and utility for 10/35 approvals (29%). In three cases (9%), PMS were were related to analytic validity and in seven cases (20%) to biomarker clinical utility. We could not find the PMS results for three drugs (denileukin diftitox, pertuzumab, and imatinib/CD177). We review here the four cases with available results.

PMS led to new or modified indications in two cases (erlotinib and crizotinib). Erlotinib was first approved in 2004 for a non–biomarker-based indication (locally advanced or metastatic NSCLC). In June 2010, a supplemental indication was granted for maintenance treatment of locally advanced or metastatic NSCLC, with a post-marketing commitment to conduct an RCT including all patients and measure endothelial growth factor receptor (EGFR) mutation status and another trial restricted to patients with EGFR mutations. Fulfillment of the latter led to a change in the indication, with restriction to patients with EGFR-mutated tumors. Similarly, the post-marketing commitment to study crizotinib in ALK-negative patients led to the discovery that many of these patients had arrangements in other markers including ROS proto-oncogene 1 (ROS1) and c-MET. Later, a clinical trial restricted to patients with ROS1-rearranged tumors led to a new approval of crizotiniub in 2016 for this condition^32^. In one case (trastuzumab), the PMS was a post-hoc analysis of the pivotal trial focusing on the dose–effect relationship between the intensity of biomarker expression and the response to the treatment, which is a strong *a posteriori* argument for having first excluded the biomarker-negative patients. There was a dose–effect relationship between the intensity of HER2 staining by immunohistochemistry and response in the two studies supporting this approval. Finally, for panitumumab, the PMS showed no link between EGFR expression intensity by immunohistochemistry and clinical benefit^33^. In the meantime, the predictive role of KRAS in this indication was discovered.

## Discussion

In this review of clinical trials supporting FDA-approved oncology drugs with required pharmacogenomic biomarker testing in their labels, we found that two thirds of FDA approvals for anticancer agents were based on trials excluding biomarker-negative patients. For the 11 other approvals that were authorized on the basis of data gathered from both biomarker-negative and -positive patients, RCT with treatment-by-biomarker interaction data were available for six approvals and were statistically significant for three.

Our findings indicate that excluding biomarker-negative patients from treatments seldom relies on clinical evidence for such exclusion but is merely the result of a drug development focused on trials including only biomarker-positive patients. Therefore, biomarker-negative patients may lose out on treatment if they might actually benefit. However, a subset of these patients may be positive for other biomarkers and, thus, candidates for alternative treatments. Therefore, evaluating the drug in biomarker-negative patients is even more difficult.

Indeed, excluding biomarker-negative patients from trials supporting drug approval depends on the confidence in the absence of effect in those patients based on the biological rationale, knowledge of the drug’s mechanism of action, preclinical data^34^, the seriousness of the disease treated (delaying approval for biomarker-positive patients is often not acceptable)^35^, the availability of an alternative treatment, the prevalence of biomarker-positive patients^35^, the confidence in the technique and in the threshold used to dichotomize patients^36^, and ethical considerations. Furthermore, the decision to include or not biomarker-negative patients in a trial could be different from the point a view of a scientist, a patient and a drug sponsor because of conflicting priorities. From the scientific perspective, the priority may be a robust study design that provides conclusive information regarding clinical utility and so to include biomarker-negative patients. However, the patient perspective could underlined the desire for access to effective medications, and the fear (and ethics) of being randomized to a drug that may be ineffective based on preliminary data. In addition, the final decision is made by the drug sponsor (possibly after a preliminary dialogue with regulatory agencies) based on marketing considerations, speed of approval process and thus the desire for efficient drug development programs that do not enroll patients unlikely to respond.

We need to have evidence from trials including biomarker-negative patients in order to be able to correctly assess the clinical utility and, if the biomarker is proven clinically useful, to confidently exclude biomarker-negative patients from treatment^12^. However, in many cases, exposing biomarker-negative patients to a potentially toxic treatment may be unethical if there are legitimate reasons to consider they would not derive benefit. In several cases, this exclusion of biomarker-negative patients from treatment may reflect a high level of pre-clinical evidence, but, in this case, patients and physicians should be aware of this assumption. However, the story of the anti-EGFR monoclonal antibody panitumumab raises the question of a too strong confidence we place in the mechanism of action^37^. Based on its mechanism of action, panitumumab was initially restricted to patients with tumors that overexpressed EGFR, but it was later shown that there was no relationship between intensity of EGFR expression and response to the treatment and that only patients with KRAS wild-type tumors could derive a benefit from the drug^38, 39^.

Moreover, predictive tests used to select patients to be treated have limitations^40^. Most biomarkers are dichotomized, and the choice of threshold and technique used to classify patients as positive and negative, for example, for the HER2 biomarker in breast cancer is uncertain^36, 40^. Furthermore, drugs may have off-target effects, and off-target trials are not rare. For example, lapatinib, a drug targeting HER2, has been tested in eight different molecularly-defined subgroups, and more than 50 trials have been conducted without the use of any biomarker^41^. Furthermore, the case of crizotinib, when attention to the efficacy in patients with ALK-negative tumors led to a new indication in ROS1-rearranged tumors, illustrates the dynamic nature of evidence and the complexity of generating and evaluating clinical utility with the increasing pace of biomarker discoveries. This situation underlines the great ethical and scientific difficulties in producing a high level of evidence of clinical utility^12^.

Importantly, all clinical studies being enriched does not mean that the clinical utility is low but that there is no clinical evidence. Moreover, some of the drugs included in this study exhibited what some have called “exceptional activity”^42^ and they have great value for biomarker-positive patients.

### Limitations

In seven cases of supplemental approvals, no medical review was available, so we used the drug label and approval letter for analysis, which likely underestimated the number of studies supporting drug approval because all studies are not reported in the label. Nonetheless, drug labels do report the most important studies and evidence supporting clinical utility, when it exists. For cetuximab/KRAS, several retrospective analyses of RCTs are reported in the label and the evidence was deemed adequate. Another potential limitation is that interaction tests are underpowered. As shown by Brookes *et al*., a trial with 80% power for the overall effect has only 29% power to detect an interaction effect of the same magnitude and at the same level^25^. However, the studies included in this review support the approval of a drug with a requirement for a predictive biomarker testing before prescription. Because of this requirement, we could expect that the differences in effect between biomarker-positive and -negative patients would have been significant even if they were not *a priori* powered to detect such an interaction. For instance, even if the studies supporting cetuximab and panitumumab were not planned to detect an interaction, the magnitude of the interaction was so great that it was detected. Furthermore, because of the reduced power of interaction tests, we chose a significance level of two-sided 0.10.

We studied the anti-cancer drugs from a US-centric point of view, mainly because other regulatory agencies do not make available an exhaustive and updated list of drugs with pharmacogenomic biomarkers in their labels as compared with the US FDA. However, cancer drugs approved by the FDA are very likely to be also approved by other agencies such as the European Medicines Agency, and drug sponsors are likely to have submitted the same studies to these other regulatory agencies^43^. Therefore, we are confident that our results are robust to these limitations.

### Conclusion

We found that the requirement of a predictive biomarker testing before prescription of an anti-cancer drug is often the result of drug development conducted in only biomarker-positive patients and seldom relies on a statistically significant treatment-by-biomarker interaction. Even though drug development in oncology will increasingly face ethical and scientific challenges, clinicians and patients should be aware of these limitations.

## Electronic supplementary material

Supplementary Information
