# Supplementary material for: Evidence for Treatment-by-Biomarker interaction for FDA-approved Oncology Drugs with Required Pharmacogenomic Biomarker Testing
Source: Sci Rep. 2017 Jul 31;7:6882. doi: 10.1038/s41598-017-07358-7 (PMC5537292; doi:10.1038/s41598-017-07358-7)
Supplement: Supplementary file 1 — Supplementary Information [file 41598_2017_7358_MOESM1_ESM.pdf]

Evidence for Treatment-by-Biomarker interaction for FDA-approved Oncology Drugs with Required Pharmacogenomic Biomarker Testing

Alexandre Vivot, Isabelle Boutron, Geoffroy Béraud-Chaulet, Jean-David Zeitoun, Philippe Ravaud, and Raphaël Porcher.

Supplementary Information

Table S1. List of indications excluded based on a high (>90%) biomarker prevalence (n= 14)

Table S2. Characteristics of the 80 clinical studies supporting approval of oncology drugs with required pharmacogenomic biomarker testing

Table S3. Characteristics of the 40 pivotal trials supporting approval of oncology drugs with required pharmacogenomic biomarker testing

Supplementary Methods. Data extraction Form.

Table S1. List of indications excluded based on a high (>90%) biomarker prevalence (n= 14).

Drug	Biomarker Gene	Indication
Arsenic Trioxide	PML/RARA	APL
Bosutinib	BCR/ABL1	Ph+ CML
Dasatinib	BCR/ABL1	Ph+ CML in chronic phase
Dasatinib	BCR/ABL1	Chronic, accelerated, or myeloid or lymphoid blast phase Ph+ CML
Imatinib	KIT	Kit (CD117)-positive unresectable and/or metastatic malignant GIST
Imatinib	KIT	Adjuvant treatment of adult patients following resection of Kit (CD117)-positive GIST
Imatinib	BCR/ABL1	Newly diagnosed adult and pediatric patients with Ph+ CML in chronic phase
Imatinib	BCR/ABL1	Patients with Ph+ CML (blast crisis, acute phase or chronic phase) after failure of interferon-alpha therapy
Nilotinib	BCR/ABL1	Ph+ CML in chronic phase
Nilotinib	BCR/ABL1	Ph+ CML chronic phase and acute phase in adult patients resistant to or intolerant to prior therapy that included imatinib
Ponatinib	BCR/ABL1	T315I-positive CML (chronic phase, accelerated phase, or blast phase) and T315I Ph+ ALL
Rituximab	MS4A1	CD20-positive B-cell non-Hodgkin's lymphoma
Tositumomab	MS4A	CD20-positive, relapsed or refractory, low-grade, follicular, or transformed non-Hodgkin's lymphoma with disease progression during or after rituximab
Tretinoin	PML/RARA	Induction of remission in patients with APL

Table S2. Characteristics of the 80 clinical studies supporting approval of oncology drugs with required pharmacogenomic biomarker testing. Data are n (%) .

Characteristic	Total	Restricted to BM+	Non-restricted	Pivotal	Non-Pivotal
No. of studies	80	54	25	40	40
Phase					
I	11 (14)	7 (13)	3 (12)	2 (5)	9 (22)
II	30 (38)	24 (44)	6 (24)	9 (22)	21 (52)
III	37 (46)	23 (43)	14 (56)	29 (73)	8 (20)
Case report	2 (3)	0 (0)	2 (8)	0 (0)	2 (5)
Enrichment (one missing value)					
Clinical	5 (6)	0 (0)	5 (20)	3 (8)	2 (5)
No	20 (25)	0 (0)	20 (80)	7 (18)	13 (33)
Yes	54 (68)	54 (100)	0 (0)	30 (75)	24 (62)
Design (one missing value)					
Single-arm with enrichment	30 (38)	29 (54)	1 (4)	6 (15)	24 (62)
Single-arm without enrichment	9 (11)	0 (0)	9 (36)	4 (10)	5 (13)
Randomized trial with enrichment	25 (32)	25 (46)	0 (0)	24 (60)	1 (3)
Randomized trial without enrichment	13 (17)	0 (0)	13 (52)	6 (15)	7 (18)
Case report	2 (3)	0 (0)	2 (8)	0 (0)	2 (5)
Primary endpoint					
Multiple	3 (4)	3 (6)	0 (0.0)	3 (8)	0 (0)
OS	3 (4)	1 (2)	2 (8)	1 (3)	2 (5)
Other	2 (3)	2 (4)	0 (0)	0 (0)	2 (5)
PFS/DFS	24 (30)	13 (24)	11 (44)	18 (45)	6 (15)
Response	45 (56)	32 (59)	12 (48)	15 (38)	30 (75)
Time to progression	3 (4)	3 (6)	0 (0)	3 (8)	0 (0)
Median [IQR] no. of patients	173 [62–572]	163 [73–468]	427 [49–865]	457 [170–724]	96 [40–185]

Table S3. Characteristics of the 40 pivotal trials supporting approval of oncology drugs with required pharmacogenomic biomarker testing.

Approval	Design	N	Endpoint
Ado-Trastuzumab Emtansine/ERBB2/HER2+ metastatic breast cancer	Phase 3 RCT	991	Multiple
Afatinib/EGFR/metastatic NSCLC	Phase 3 RCT	345	PFS/DFS
Anastrozole/ESR1, PGR/adjuvant treatment of postmenopausal women with HR + early breast cancer	Phase 3 RCT	9366	PFS/DFS
Anastrozole/ESR1, PGR/first-line treatment of postmenopausal women with HR + or HR unknown locally advanced or metastatic breast cancer	Phase 3 RCT	668	Multiple
Cetuximab/EGFR/EGFR-expressing colorectal cancer	Phase 2 RCT	329	Response
Cetuximab/KRAS/EGFR-expressing, KRAS-mutation negative colorectal cancer	Phase 3 RCT	572	PFS/DFS
Crizotinib/ALK/metastatic NSCLC	Phase 1 Single-arm trial	154	Response
	Phase 2 Single-arm trial	148	Response
Dabrafenib/BRAF/BRAF V600E mutation-positive unresectable or metastatic melanoma as a single agent	Phase 3 RCT	250	PFS/DFS
Dabrafenib/BRAF/BRAF V600E or V600K mutation-positive unresectable or metastatic melanoma combined with trametinib	Phase 3 RCT	162	Response
Denileukin Diftitox/IL2RA/persistent or recurrent cutaneous T-cell lymphoma with malignant cells expressing the CD25 component of the IL-2 receptor	Phase 3 RCT	71	Response
Erlotinib/EGFR/first-line treatment of patients with metastatic NSCLC	Phase 3 RCT	173	PFS/DFS
Everolimus/ERBB2/HER2-negative breast cancer (advanced HR+ BC) combined with exemestane after failure of treatment with letrozole or anastrozole	Phase 3 RCT	724	PFS/DFS
	Phase 3 RCT	724	PFS/DFS
Exemestane/ESR1/adjuvant treatment of postmenopausal women with estrogen receptor-positive early breast cancer	Phase 3 RCT	4724	PFS/DFS
Fulvestrant/ESR1/hormone receptor-positive metastatic breast cancer	Phase 3 RCT	473	PFS/DFS
	Phase 3 RCT	451	PFS/DFS
Imatinib/KIT/aggressive systemic mastocytosis without the D816V c-Kit mutation or with c-Kit mutational status unknown	Phase 2 Single-arm trial	5	Response
Imatinib/BCR/ABL1/adult patients with relapsed or refractory (Ph+ ALL)	Phase 2 Single-arm trial	48	Response
Imatinib/BCR/ABL1/pediatric patients with newly diagnosed (Ph+ ALL) combined with chemotherapy	Phase 3 Single-arm trial	50	PFS/DFS

Imatinib/PDGFRB/adult patients with MDS/MPD associated with PDGFR gene re-arrangements	Phase 2 Single-arm trial	7	Response
Lapatinib/ERBB2/combined with capecitabine for treating patients with advanced or metastatic breast cancer overexpressing HER2 and who have received prior therapy	Phase 3 RCT	324	TTP
Lapatinib/ERBB2/combined with letrozole for treating postmenopausal women with HR+ metastatic breast cancer expressing HER2 receptor for whom hormonal therapy is indicated	Phase 3 RCT	1286	PFS/DFS
Letrozole/ESR1, PGR/adjuvant treatment for early breast cancer	Phase 3 RCT	5187	PFS/DFS
Letrozole/ESR1, PGR/first- and second-line treatment for advanced breast cancer	Phase 3 RCT	551	Response
	Phase 3 RCT	555	Response
	Phase 3 RCT	907	TTP
Panitumumab/EGFR/EGFR-expressing metastatic colorectal carcinoma	Phase 3 RCT	463	PFS/DFS
Pertuzumab/ERBB2/metastatic breast cancer	Phase 3 RCT	808	PFS/DFS
Pertuzumab/ERBB2/neoadjuvant treatment for breast cancer	Phase 2 RCT	417	Response
Trametinib/BRAF/unresectable or metastatic melanoma with BRAF V600E or V600K mutation	Phase 3 RCT	322	PFS/DFS
Trastuzumab/ERBB2/metastatic breast cancer	Phase 3 RCT	469	TTP
	Phase 2 Single-arm trial	222	Response
Trastuzumab/ERBB2/adjuvant therapy for breast cancer	Phase 3 RCT	1944	PFS/DFS
	Phase 3 RCT	2043	PFS/DFS
Trastuzumab/ERBB2/metastatic gastric or gastroesophageal junction adenocarcinoma	Phase 3 RCT	594	OS
Vemurafenib/BRAF/unresectable or metastatic melanoma with BRAF V600E mutation	Phase 3 RCT	675	Multiple
Ceritinib/ALK/ALK-positive metastatic NSCLC with disease progression or intolerance to crizotinib	Phase 1 Single-arm trial	246	Response
Lenalidomide/del (5q)/transfusion-dependent anemia due to low- or intermediate-1-risk myelodysplastic syndromes associated with a deletion 5q cytogenetic abnormality	Phase 2 Single-arm trial	43	Response
	Phase 2 Single-arm trial	148	Response

Level of evidence of studies in FDA submission

*Obligatoire

Identification of drug, biomarker and study

Pair.Num *

ID of drug/biomarker combinaison

Veuillez saisir un nombre supérieur à 0.

Cette question est obligatoire.

Indication

Only if there are more than one indication for the drug

Cette question est obligatoire.

Indication.Num

ID of of indication/drug/biomarker combination

Veuillez saisir un nombre supérieur à 0.

Cette question est obligatoire.

Name or ID of study

In FDA review

Cette question est obligatoire.

ClinicalTrials.gov Identifier

Cette question est obligatoire.

Status of Trial

- ○ Published
- ○ Not published, but results available elsewhere
- ○ Terminated but no results available
- ○ Ongoing

Cette question est obligatoire.

Type of study

According to FDA statement; cf Downing Jama for definition

- ○ Pivotal Study
- ○ Supportive Study
- ○ Only safety data included
- ○ Other or NA

Cette question est obligatoire.

Article Name

Author Year Journal. State all articles if necessary.

Cette question est obligatoire.

Biomarker-based Criteria for inclusion in the study

Cette question est obligatoire.

Other Methods

Phase of trial

Cette question est obligatoire.

Comparator

- ○ Actively controlled
- ○ Placebo controlled
- ○ Uncontrolled

Cette question est obligatoire.

Primary Endpoint

- ○ Overall Survival (OS)
- ○ Disease Free Survival (DFS)
- ○ Progression Free Survival (PFS)
- ○ Response Rate (RECIST Criteria)
- ○ Major Cytogenetic Response (MCyR)
- ○ Complete Cytogenetic Response (CCyR)
- ○ Overall Response Rate
- ○ Major Hematologic Response (MaHR)
- ○ Autre :

Cette question est obligatoire.

Total number of patients randomized

or treated if there is no randomization

Veuillez saisir un nombre supérieur à 0.

Cette question est obligatoire.

Biomarker assesment

Use of biomarker

- ○ Prior to the initiation of study
- ○ Retrospectively assesed

Cette question est obligatoire.

Technique to measure biomarker

Cette question est obligatoire.

Design and level of evidence

Design *

Adaptated from Tajik and Simon and Mandrekar and Sargent

- ○ 1a Single-arm with enrichment
- ○ 1b Single-arm without enrichment
- ○ 2 Comparative Study, with enrichment
- ○ 3 Comparative Study, without enrichment
- ○ 4a Biomarker-strategy with biomarker measurement in the control arm
- ○ 4b Biomarker-strategy without biomarker measurement in the control arm
- ○ 4c Biomarker-strategy with treatment randomization measurement in the control arm
- ○ 5 Combination of patients flows
- ○ 6 Case report
- ○ NA

Cette question est obligatoire.

Level of evidence determination *

From Simon et al, JNCI 2009

- A. Prospective experimental
- B. Prospective using archived samples
- C. Prospective observational
- D. Retrospective observational
- NA

Cette question est obligatoire.

Comments

Any comments

Cette question est obligatoire.

Rationale for using enrichment design

Cette question est obligatoire.

Envoyer
